# Analysis of Variation in the Origin of the Obturator Artery in Midwestern American Donor Bodies

**DOI:** 10.7759/cureus.53650

**Published:** 2024-02-05

**Authors:** Corey Diemer, Brynn Schubert, Sara Funk, Shanu Markand

**Affiliations:** 1 Anatomy, A.T. Still University, Kirksville, USA

**Keywords:** pelvic vasculature, pelvic surgery, pelvis, anatomical variation, obturator artery

## Abstract

The obturator artery (OA) is typically a branch of the anterior division of the internal iliac artery. However, an aberrant obturator artery origin may lead to clinical complications. Because of its location in the pelvic cavity, the OA is at high risk of injury or laceration during a variety of pelvic surgeries. Regarding this, variations in the origins of the OA may result in bleeding that can often be overlooked, rendering treatment ineffective. Our study aimed to assess the origins and course of the OA in Midwestern American donor bodies. Sixty-two donor bodies were obtained from the Gift of Body Donation Program at A.T. Still University’s Kirksville College of Osteopathic Medicine. The origin of each OA was documented and photographed. The OA was identified by observing the vessel's passage through the obturator foramen. Of 132 OAs studied, 72 (54.5%) had an aberrant OA. Further, 22 (16.7%) had an aberrant OA origin from the inferior epigastric artery, 20 (15.2%) had an aberrant OA origin from the posterior division of the internal iliac artery, 22 (16.7%) had an aberrant OA origin from dual origins of the anterior division of the internal iliac artery and the inferior epigastric artery, and eight (6.1%) had other aberrant OA origins. Overall, our results indicated anatomical variations are common in the origins and course of the OA. These data highlight the importance of considering variations in the OA and the prevalence of those variations during vascular and orthopedic procedures.

## Introduction

Anatomy of the obturator artery

The obturator artery (OA) is a terminal branch of the abdominal aorta within the pelvic ring. The abdominal aorta bifurcates into the right and left common iliac arteries [1]. Each common iliac artery typically branches into internal and external iliac arteries [1]. External iliac arteries (EIAs) provide the main blood supply to each lower limb [1]. The internal iliac arteries (IIAs) and their branches supply blood to the pelvis, perineum, and gluteal regions [1]. The IIA is further divided into anterior and posterior divisions, with the OA typically originating from the anterior division [1]. The OA travels through the obturator foramen with the obturator nerve and supplies the iliac bone (nutrient branch), obturator externus, iliacus, adductor magnus, adductor minimus, adductor longus, adductor brevis, pectineus, and gracilis muscles [1]. 

Variation in the obturator artery

Human anatomy studies suggest variations in the origin of the OA are common [1-10]. An aberrant obturator artery (AOA) is a vessel of an atypical origin and location. Specifically, the OA does not originate from the anterior division of the IIA. When AOAs are found, the most common origin is the inferior epigastric artery or the EIA [1,5]. One potentially fatal variation occurs when an anastomosis forms between the OA and the inferior epigastric artery so that the artery courses over the superior pubic ramus [11]. As suggested by its name of corona mortis or “crown of death,” this presentation causes an increased risk of ligation or injury during surgical interventions or pelvic fractures. Previous studies have suggested a lack of awareness and familiarity with AOA are associated with potentially life-threatening outcomes during clinical procedures [2,12]. However, these studies utilized limited donor bodies to investigate AOA. More studies with larger sample sizes are necessary to characterize the occurrence of AOAs across United States demographic groups. Further, there is paucity in the literature regarding the prevalence of AOAs in the Midwestern American population. To address these limitations, our study aimed to assess the origins and course of the OA in Midwestern American donor bodies.

## Materials and methods

All procedures in the current study were considered exempt by the A.T. Still University-Kirksville Institutional Review Board. The current study was part of a Gross Anatomy dissection course. It used 62 donor bodies of Midwestern Americans from the Gift of Body program at A.T. Still University’s Kirksville College of Osteopathic Medicine. The average age was 74.71 years old, while the range was 29-98 years old. Exclusion criteria for selecting donor bodies were autopsy, donating major organs, gross obesity, destructive trauma, extensive surgery, and communicable disease. The donor completed a brief medical history, or the next-of-kin donation filled out the medical history at the time of death. Five pelvic halves (one female, four male) were excluded from the study due to extensive trauma from previous pelvic surgery or previous dissection, resulting in the destruction of the OA and associated structures. OAs from 121 pelvic halves (69 female and 52 male) were dissected. The dissections were performed using donor bodies in the spring of 2022 and 2023. Dissections from the spring of 2022 studied 64 total OAs (32 female OAs, 32 male OAs) from 29 female and 30 male pelvic halves. Dissections from the spring of 2023 studied 68 total OAs (45 female OAs, 23 male OAs) from 40 female and 22 male pelvic halves. The initial discovery of the OA was made by Gross Anatomy students. To assess the origins and course of the OA, additional dissection of donor bodies beyond course requirements was performed by the authors specifically to reveal the OA and associated structures. Each donor body's origin and course of each OA were identified as they passed through the obturator foramen and were documented and photographed using an iPhone 12 Pro camera. Frequency and percentage (%) were used to summarize OA variation findings overall by sex and by left and right pelvic halves.

## Results

Table 1 summarizes our comparisons of the origins of the OA between female and male donor bodies in the left and right pelvic halves. The prevalence of normal origins of OA anatomy was more prevalent in male donor bodies (left = 70.4%, right = 57.1%). Female donor bodies more often had aberrant anatomy (67.5%), where the OA originated from the IIA posterior division (19.5%), the inferior epigastric artery (20.8%), the corona mortis presentation (20.8%) or other origins (6.5%). In female and male donors, percent (%) were calculated relative to the right or left hemipelvis. Raw data are available in the appendix.

**Table 1:** Comparisons of the obturator artery (OA) origins between female and male donor bodies in left and right pelvic halves. OA origins between female and male donor bodies are represented as number (n) of OAs and percent (%) in left and right pelvic halves. Corona mortis represented the presence of two OAs in one pelvic half, originating from both the anterior division of the internal iliac artery and the inferior epigastric artery. Other OA origins include the posterior division of the superior gluteal artery, the external iliac artery, and the inferior vesicle artery.

OA Origin	No. (%) Female			No. (%) Male		
	Total (n=77)	Left (n=38)	Right (n=39)	Total (n=55)	Left (n=27)	Right (n=28)
Anterior division of the internal iliac artery	25 (32.5)	11 (29.0)	14 (35.9)	35 (63.6)	19 (70.4)	16 (57.1)
Posterior division of the internal iliac artery	15 (19.5)	8 (21.1)	7 (18.0)	5 (9.1)	2 (7.2)	3 (10.7)
Inferior epigastric artery	16 (20.8)	9 (23.7)	7 (18.0)	6 (10.9)	3 (11.1)	3 (10.7)
Corona Mortis	16 (20.8)	8 (21.1)	8 (20.5)	6 (10.9)	2 (7.4)	4 (14.3)
Other	5 (6.5)	2 (5.3)	3 (7.7)	3 (5.5)	1 (3.7)	2 (7.1)

**Figure 1:**
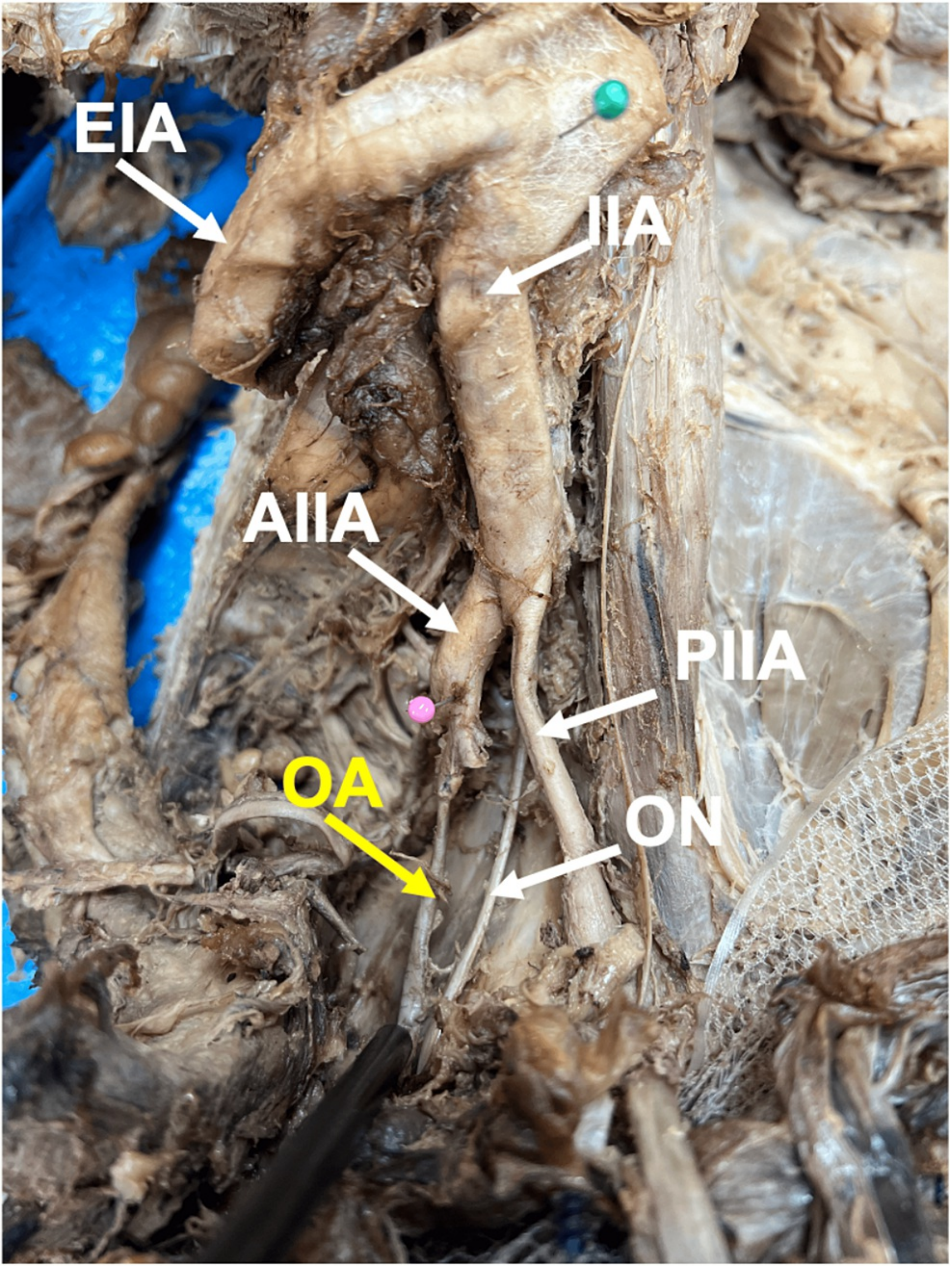
Typical anatomy of the obturator artery (OA, yellow) originating from the anterior division of the internal iliac artery (AIIA, pink pin). As shown, the OA anatomy typically originates from the anterior division of the IIA; the OA (yellow label) arises from the anterior division of the IIA (pink pin indicates AIIA). The image is from a male donor body, right hemipelvis. Abbreviations: IIA, internal iliac artery; EIA, external iliac artery; ON, obturator nerve; PIIA, posterior division of the IIA

Figure 1 presents the typical origin and course of the OA. Of the 132 OAs dissected during the study, 60 (45.5%) had normal anatomy, where the OA originated from the anterior division of the IIA. Seventy-two (54.5%) of the 132 had AOA origins. Of the 60 (45.5%) normal OA origins, 25 (18.9%) OA origins were from female donors, and 35 (26.5%) OA origins were from male donors. Percents (%) were calculated relative to total OAs in the study. 

**Figure 2:**
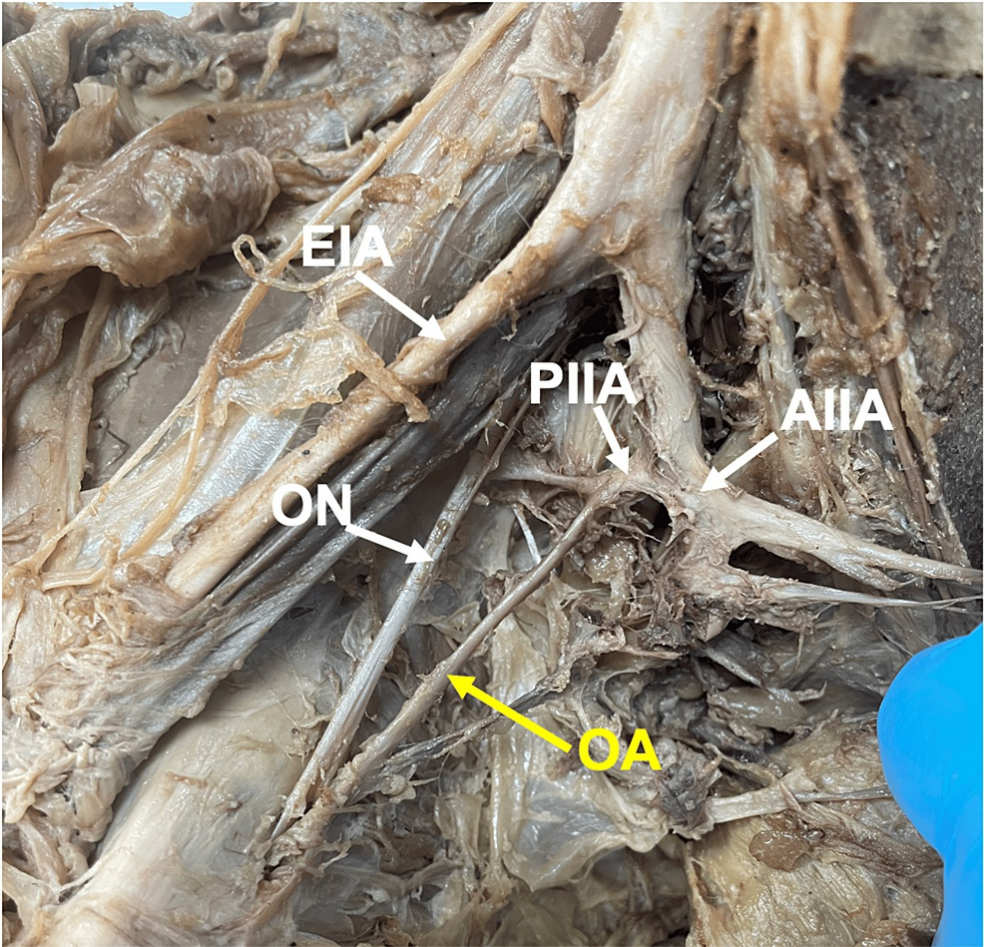
The obturator artery (OA) originates from the posterior division of the internal iliac artery (PIIA). As shown, the anterior and posterior divisions of the IIA originated from the IIA. Specifically, the OA (yellow label) originates from the posterior division of the IIA. The image is from a female donor body, right hemipelvis. Abbreviations: IIA, internal iliac artery; AIIA, anterior division of IIA; EIA, external iliac artery; ON, obturator nerve

Twenty (15.2%) OAs originated from the posterior division of the IIA (Figure 2). Of the 20 (15.2%) OAs originating from the posterior division of the IIA, 15 (11.4%) OA origins were from female donors, and five (3.8%) OA origins were from male donors. Percents (%) were calculated relative to total OAs in the study. 

**Figure 3:**
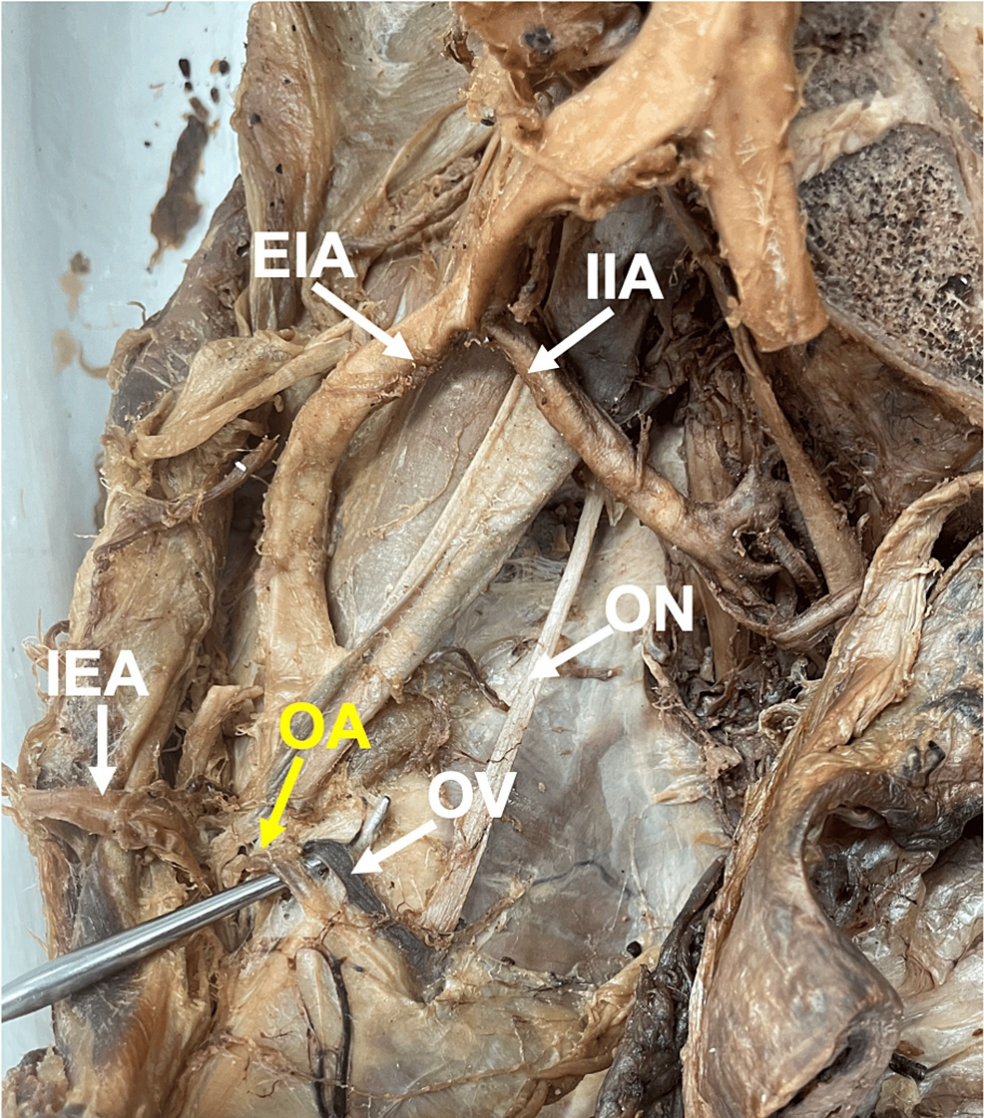
Aberrant obturator artery (OA) originating from the inferior epigastric artery (IEA). The OA (yellow label and a probe) originates from the IEA. As the probe indicates, OA is accompanied by the obturator vein (OV). The image is from a female donor body, right hemipelvis. Abbreviations: IIA, internal iliac artery; EIA, external iliac artery; ON, obturator nerve

Twenty-two (16.7%) originated from the inferior epigastric artery (Figure 3). Of the 22 (16.7%) OAs originating from the inferior epigastric artery, 16 (12.1%) OA origins were from female donors, and six (4.6%) OA origins were from male donors. Percents (%) were calculated relative to total OAs in the study. 

**Figure 4:**
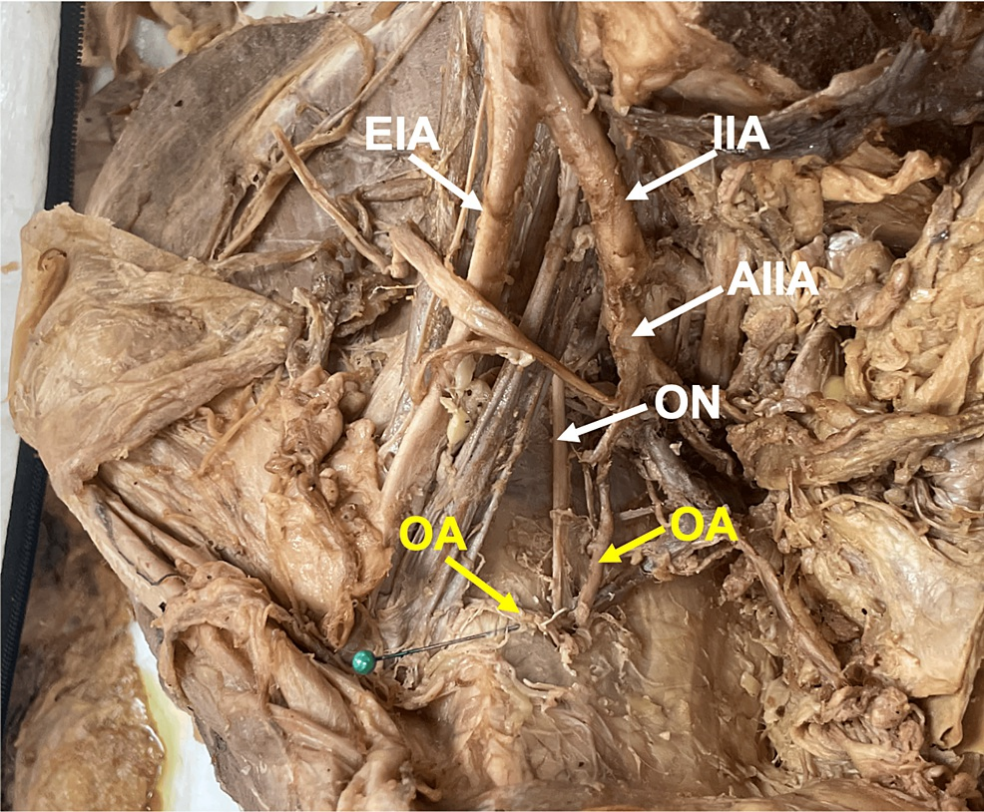
Dual aberrant obturator arteries (OAs) originate from the anterior division of the internal iliac artery (AIIA) and the inferior epigastric artery. Dual OAs (yellow labels) were observed in 11 pelvic halves. The first OA had typical anatomy originating from the anterior division of the IIA. The second OA is marked with a green pin and originated from the inferior epigastric artery. The image is from a female donor body, left hemipelvis Abbreviations: IIA, internal iliac artery; EIA, external iliac artery; ON, obturator nerve.

Twenty-two (16.7%) OAs originated from the IIA anterior division and inferior epigastric artery (Figure 4), presenting with corona mortis. Of the 22 (16.7%) corona mortis origins, 16 (12.1%) were from female donors, and six (4.6%) were from male donors. Percents (%) were calculated relative to total OAs in the study. 

Eight (6.1%) OAs arose from other origins, including the EIA, the posterior division of the superior gluteal artery, and the inferior vesicle artery. Of the eight (6.1%) other origins, five (3.8%) OA origins were from female donors, and three (2.3%) OA origins were from male donors. Percents (%) were calculated relative to total OAs in the study. 

## Discussion

Anatomical variations in OA are widespread [1,3,4,6,11,13-15] and may have a major impact on clinical diagnosis, interventions, and patient safety. Therefore, understanding the prevalence of OA variations and their associated complications is necessary to enhance diagnostic studies, preoperative planning, operative decisions, and overall surgical safety, particularly for procedures in the pelvic region. Of our study's 62 Midwestern American donor bodies, we observed aberrant OA origins in 54.5% of pelvic halves.

Previous studies have reported AOA origins with a prevalence of 22%-60% [1,3,4,6,11,13,14]. These findings underscore the likelihood of encountering AOA during operative procedures. For instance, Granite et al. [1] found that 38.9% of cadavers in their study had an anomalous OA origin, and some had bilateral anomalous origins, with 33% originating from the inferior epigastric artery and 5.6% from the EIA. A study by Rajive and Pillay [15] reported additional aberrant origins that were unobserved in our study. Specifically, they found OAs originating from the inferior epigastric artery, from both the IIA and EIA, and the posterior trunk of the IIA, superior gluteal artery, inferior gluteal artery, and pudendal artery [15]. We observed similar variations in our study, especially OA arising from the inferior epigastric and the posterior division of the internal iliac. Studies also support the prevalence of AOA and emphasize sex-specific differences in findings [4,13,15]. In our study, female donor bodies had AOA more often than males bilaterally.

The clinical importance of recognizing vascular variations, particularly about unknown or unidentifiable bleeding during pelvic injuries or procedures, is well documented [1,6,11,15]. In a meta-analysis of studies from 1952-2020, Brachini et al. [16] reported that an average of 22.3% of patients had AOA. In another study, Perandini et al. [11] reported a prevalence of corona mortis due to AOA in 40.6% of 150 patients, highlighting the importance of surgeon awareness and preoperative assessment. We report 16.7% of 132 OA with corona mortis presentation. Notably, corona mortis, which involves an anastomosis between the OA and inferior epigastric artery, increases the risk of surgical complications [17-20]. Therefore, the potential for life-threatening bleeding underscores the importance of identifying OA variations that lead to corona mortis [2,11,15,17,18,20]. 

As such, some have advocated for preoperative diagnostic tools to enhance patient safety and reduce surgical complications. For example, Brachini et al. [16] recommended radiological examination to determine pelvic vasculature patterns that could contribute to improved patient care. Similarly, Requarth and Miller [4] showed that computed tomography angiography had high sensitivity for identifying AOA origins. Others have measured OA diameters and reported significant variability that ranged from 1.63 mm to 28.3 mm [11,19,20]. In their study investigating different aspects of OA anatomy, Sañudo et al. [14] noted variations in the number of OA origins, where 96.55% of OA had a single origin, 3.02% had a double origin, and 0.43% had a triple origin. These studies, including our own, reflect the importance of exploring AOA findings in ways unique to most OA studies thus far. The more variability in OA origins is understood, the more preparation can be applied to surgical cases.

Strengths of our study include a sample size of 62 donor bodies, high-quality images, inclusion of both sexes, and wider age range (29-98 years old). Limitations of this study include a sample population from a limited geographical location, a lack of other live imaging modalities, and a lack of data on the length and diameter of the OAs as we focused on origins only. Future studies will explore including different imaging modalities to supplement data obtained from donor bodies.

## Conclusions

The current study assessed the origins and course of the OA in Midwestern American donor bodies. Fifty-four percent of OAs were aberrant, with the most common variation being an inferior epigastric and posterior division of the internal iliac artery. AOAs were more common in females than males. Our data highlighted anatomical variations in the origin of the OA. These variations may have important surgical implications for interventions of the pelvis, the risk of hemorrhage from pelvic fractures, and overall patient safety. Therefore, surgeon awareness of the prevalence of these variations is critical in preventing or stopping pelvic bleeds during surgery. Further, familiarity with variations in OA origin may have implications for vascular and orthopedic surgeons.

## Appendices

**Table 2:** Raw Data from Year 1 and Year 2 (total and percentage) This table shows the total numbers and percents of obturator artery (OA) origins from this study. "Other" category was further broken down by different origins and by sex.

Year 1 and 2 Data		
	Total	Percent
OA origins	132	100.0%
inferior epigastric	22	16.7%
internal iliac post division	20	15.2%
internal iliac ant division	60	45.5%
internal iliac ant division + inferior epigastric (CM)	22	16.7%
other	8	6.1%
Typical OA origin anatomy	60	45.5%
Aberrant OA origin anatomy	72	54.5%
"Other" breakdown:	Total	Percent
inferior vesicle artery	1	1%
external iliac	5	4%
superior gluteal post division	2	2%
"Other breakdown" male vs. female	Male	Female
inferior vesicle artery	0	1
external iliac	2	3
superior gluteal post division	1	1
total	3	5
percent (%)	2.27	3.79

**Table 3:** Raw Data of Obturator Artery (OA) Origins from Year One (2022) and Year Two (2023) Organized by Sex and Left or Right Hemipelvis This table shows the OA origins and comments gathered from year one and two of the study. The table is organized based on donor body sex, and the origins of the OAs examined in their left and right hemipelvises. Comments were made based on exclusion criteria, damage to the OAs and surrounding structures, or general observations. IIA, internal iliac artery; IEA, inferior epigastric artery

OA Origins from Year 1 and Year 2				
Year 1 Data: 2022				
Table	Sex	Left	Right	Comments
1	Male	Inferior epigastric	internal iliac ant division	
2	Female	Internal iliac post division	internal iliac ant division	left cut, still visible
3	Male	internal iliac ant division	internal iliac ant division	
4	Female	inferior epigastric	internal iliac ant division, inferior epigastric	right dual OAs
5	Male	external iliac	external iliac	
6	Female	internal iliac post division	external iliac	
7	Male	internal iliac ant division	internal iliac ant division	
8	Female		internal iliac ant division	left cut, exclude
9	Male	internal iliac ant division	internal iliac ant division	
10	Female	internal iliac post division	internal iliac ant division, inferior epigastric	right dual OAs
11	Male	internal iliac ant division	internal iliac ant division	
12	Female	inferior epigastric	internal iliac ant division	
13	Male	internal iliac ant division	superior gluteal post division	
14	Female	internal iliac ant division	external iliac	
15	Male	inferior epigastric	inferior epigastric	
16	Female	superior gluteal post division	external iliac	
17	Male	internal iliac post division	internal iliac post division	
18	Female	inferior epigastric	internal iliac ant division	
19	Male	internal iliac ant division	internal iliac ant division	
20	Female	inferior epigastric	inferior epigastric	
21	Male			poor dissection, undetermined- exclude
22	Female	internal iliac ant division	internal iliac ant division	
23	Male	internal iliac ant division	internal iliac ant division	
24	Female	internal iliac ant division, inferior epigastric	internal iliac post division	
25	Male	internal iliac ant division	internal iliac ant division, inferior epigastric	
26	Female	internal iliac post division	internal iliac ant division	
27	Male	internal iliac ant division, inferior epigastric	internal iliac ant division	
28	Female	internal iliac ant division	internal iliac ant division	
29	Male	internal iliac ant division	internal iliac post division	
30	Female	internal iliac ant division	inferior epigastric	
31	Male	internal iliac ant division	internal iliac ant division	
Year 2 Data: 2023				
1	Male		internal iliac ant division	L IIA is broken, exclude
2	Female	internal iliac ant division, inferior epigastric	internal iliac post division	
3	Male	internal iliac ant division	internal iliac post division	
4	Female	internal iliac ant division	internal iliac post division	
5	Male	internal iliac ant division		R vessels are gone-exclude
6	Female	inferior epigastric	internal iliac ant division, inferior epigastric	a lot cut on left side, but IEA
7	Male	internal iliac ant division	internal iliac ant division	
8	Female	internal iliac ant division	internal iliac ant division, inferior epigastric	
9	Female	inferior epigastric	internal iliac post division	
10	Female	inferior vesicle artery	inferior epigastric	L branch from bladder giving rise to OA
11	Male	internal iliac ant division	inferior epigastric	
12	Female	internal iliac ant division	internal iliac ant division	No ID tag
13	Male	internal iliac ant division	internal iliac ant division, inferior epigastric	
14	Female	internal iliac post division	internal iliac post division	IIA stent
15	Male	internal iliac ant division	internal iliac ant division	
16	Female	inferior epigastric	internal iliac post division	
17	Male	internal iliac ant division	internal iliac ant division	
18	Female	internal iliac post division	inferior epigastric	
19	Female	internal iliac ant division	inferior epigastric	
20	Female	internal iliac ant division, inferior epigastric	inferior epigastric	origin cut from IIA on L side, still visible
21	Female	internal iliac ant division	internal iliac ant division	
22	Female	internal iliac post division	internal iliac ant division	R cut, still visible
23	Male	inferior epigastric	inferior epigastric	
24	Female	inferior epigastric	internal iliac ant division	
25	Male	internal iliac ant division	internal iliac ant division	
26	Female	internal iliac ant division	internal iliac ant division	
27	Male	internal iliac post division	internal iliac ant division	
28	Female	inferior epigastric	internal iliac ant division	
29	Male	internal iliac ant division	internal iliac ant division	
30	Female	internal iliac ant division	inferior epigastric	
31	Female	interior iliac post division	interior iliac post division	
32	Female	internal iliac ant division, inferior epigastric	internal iliac ant division	dual supply on L side

**Table 4:** Total Obturator Arteries (OAs) Studied Broken Down by Year, by Sex, and by Left or Right Hemipelvis This table organizes total OAs studied by year of study, by number of pelvic halves, and by number of OAs, then further brokendown by sex of donor. Data from each year was then combined to find total OAs studied and total pelvic halves examined for each sex of donor.

Year 1 and Year 2 Data: Female vs. Male Arteries	
Year 1 Data: 2022	
total pelvic halves	59
total arteries	64
female pelvic halves	29
female arteries total	32
female arteries left	15
female arteries right	17
male pelvic halves	30
male arteries total	32
male arteries left	16
male arteries right	16
Year 2 Data: 2023	
total pelvic halves	62
total arteries	68
female pelvic halves	40
female arteries total	45
female arteries left	23
female arteries right	22
male pelvic halves	22
male arteries total	23
male arteries left	11
male arteries right	12
Year 1 and 2 Combined Data	
total pelvic halves	121
total arteries	132
female pelvic halves	69
female arteries total	77
female arteries left	38
female arteries right	39
male pelvic halves	52
male arteries total	55
male arteries left	27
male arteries right	28

**Table 5:** Raw Data of Obturator Artery (OA) Origins of Females vs. Males by Total Numbers and Percents The table shows the raw data of all OA origins from female and male donors. These data are organized first by female and male OA origins compared to total OAs studied, then by female and male OA origins compared to total female and male OA origins studied (non-sex specific percents, then sex-specific percents).

Year 1 and 2 Data: Female vs Male						
Percents relative to total arteries: Female						
	Total	% Total	Left	% Left	Right	% Right
Female	77	58.33%	38	69.09%	39	70.91%
inferior epigastric	16	12.12%	9	16.36%	7	12.73%
internal iliac post division	15	11.36%	8	14.55%	7	12.73%
internal iliac ant division	25	18.94%	11	20.00%	14	25.45%
CM	16	12.12%	8	14.55%	8	14.55%
other	5	3.79%	2	3.64%	3	5.45%
Percents relative to total arteries: Male						
	Total	% Total	Left	% Left	Right	% Right
Male	55	41.67%	27	35.06%	28	36.36%
inferior epigastric	6	4.55%	3	3.90%	3	3.90%
internal iliac post division	5	3.79%	2	2.60%	3	3.90%
internal iliac ant division	35	26.52%	19	24.68%	16	20.78%
CM	6	4.55%	2	2.60%	4	5.19%
other	3	2.27%	1	1.30%	2	2.60%
Percents relative to total female OAs & left/right OA origins						
	Total female	% Total female	Left	% Left	Right	% Right
Female	77	100.00%	38	100.00%	39	100.00%
inferior epigastric	16	20.78%	9	23.68%	7	17.95%
internal iliac post division	15	19.48%	8	21.05%	7	17.95%
internal iliac ant division	25	32.47%	11	28.95%	14	35.90%
CM	16	20.78%	8	21.05%	8	20.51%
other	5	6.49%	2	5.26%	3	7.69%
Female						
Typical OA origin	AOA origin					
32.47%	67.53%					
Percents relative to total male OAs & left/right OA orgins						
	Total Male	% Total Male	Left	% Left	Right	% Right
Male	55	100.00%	27	100.00%	28	100.00%
inferior epigastric	6	10.91%	3	11.11%	3	10.71%
internal iliac post division	5	9.09%	2	7.41%	3	10.71%
internal iliac ant division	35	63.64%	19	70.37%	16	57.14%
CM	6	10.91%	2	7.41%	4	14.29%
other	3	5.45%	1	3.70%	2	7.14%
Male						
Typical OA origin	AOA origin					
63.64%	36.36%					

## References

[1] Granite G, Meshida K, Wind G (2020). Frequency and clinical review of the aberrant obturator artery: a cadaveric study. Diagnostics (Basel).

[2] Sambhav K, Nayyar AK, Elhence A, Gupta R, Ghatak S (2022). Anatomical variations of corona mortis in the anterior intrapelvic approach: a cadaveric study. Mymensingh Med J.

[3] Gilroy AM, Hermey DC, DiBenedetto LM, Marks SC Jr, Page DW, Lei QF (1997). Variability of the obturator vessels. Clin Anat.

[4] Requarth JA, Miller PR (2011). Aberrant obturator artery is a common arterial variant that may be a source of unidentified hemorrhage in pelvic fracture patients. J Trauma.

[5] Bosse BL, Palacios VJ, Dutcher DW, Etter EJ, Lim PC, Cobine CA, Moritz GL (2022). Unconventional obturator artery nutrient branch: image of an anatomical variation. Diagnostics (Basel).

[6] Sonje PD, Vatsalaswamy P (2016). Study of variations in the origin of obturator artery. Indian J Vasc Endovasc Surg.

[7] Nayak SB, Guru A, Reghunathan D, Maloor PA, Padavinangadi A, Shantakumar SR (2016). Clinical importance of a star shaped branch of internal iliac artery and unusual branches of an abnormal obturator artery: rare vascular variations. J Vasc Bras.

[8] Nayak SB, Shetty SD, Shetty P (2014). Presence of abnormal obturator artery and an abnormal venous plexus at the anterolateral pelvic wall. OA Case Rep.

[9] Goke K, Pires LA, Leite TF, Chagas CA (2016). Rare origin of the obturator artery from the external iliac artery with two obturator veins. J Vasc Bras.

[10] Kumar S, Minz S (2017). A study of variations in the origin of obturator artery in the human cadavers and its clinical significance. Natl J Clin Anat.

[11] Perandini S, Perandini A, Puntel G, Puppini G, Montemezzi S (2018). Corona mortis variant of the obturator artery: a systematic study of 300 hemipelvises by means of computed tomography angiography. Pol J Radiol.

[12] Yamaki K, Saga T, Doi Y, Aida K, Yoshizuka M (1998). A statistical study of the branching of the human internal iliac artery. Kurume Med J.

[13] Pai MM, Krishnamurthy A, Prabhu LV, Pai MV, Kumar SA, Hadimani GA (2009). Variability in the origin of the obturator artery. Clinics (Sao Paulo).

[14] Sañudo JR, Mirapeix R, Rodriguez-Niedenführ M, Maranillo E, Parkin IG, Vázquez T (2011). Obturator artery revisited. Int Urogynecol J.

[15] Rajive AV, Pillay M (2015). A study of variations in the origin of obturator artery and its clinical significance. J Clin Diagn Res.

[16] Brachini G, Matteucci M, Sapienza P (2023). Systematic review and meta-analysis of the variants of the obturatory artery. J Clin Med.

[17] Marsman JW, Schilstra SH, van Leeuwen H (1984). Angiography and embolization of the corona mortis (aberrant obturator artery). A source of persistent pelvic bleeding. Rofo.

[18] Koh M, Markovich B (2019). Anatomy, abdomen and pelvis, obturator nerve. StatPearls.

[19] Beya R, Jérôme D, Tanguy V (2023). Morphodynamic study of the corona mortis using the SimLife(®) technology. Surg Radiol Anat.

[20] Heichinger R, Pretterklieber ML, Hammer N, Pretterklieber B (2023). The corona mortis is similar in size to the regular obturator artery, but is highly variable at the level of origin: an anatomical study. Anat Sci Int.

